# Profile of bacterial meningitis pathogens circulating in a cohort of school children in Côte d’Ivoire using 16S rRNA gene sequencing

**DOI:** 10.1186/s12879-026-13880-9

**Published:** 2026-06-30

**Authors:** Kolotioloman Jérémie Tuo, Kanny Diallo, Tiémélé Laurent-Simon Amoikon, Kouassi Firmin Missa, Kossia Debia Thérèse Gboko, Biego Guillaume Gragnon, Joyce Mwongeli Ngoi, Robert J. Wilkinson, Gordon Awandare, Bassirou Bonfoh, Adolphe Zézé

**Affiliations:** 1 Centre Suisse de Recherches Scientifiques en Côte d’Ivoire (CSRS), Abidjan, Côte d’Ivoire; 2 Laboratoire de Microbiologie, Biotechnologies et Bioinformatique, Unité Mixte de Recherche et d’Innovation en Sciences Agronomiques et Procédés de Transformation, Institut National Polytechnique Félix Houphouët-Boigny (INP-HB), Yamoussoukro, Côte d’Ivoire; 3 West African Centre for Cell Biology of Infectious Pathogens (WACCBIP), University of Ghana, Accra, Ghana; 4 Institut Pierre Richet de Bouaké, Institut National de Santé Publique (INSP), Abidjan, Côte d’Ivoire; 5 Laboratoire de Biologie et Santé, UFR Biosciences, Université Félix Houphouët Boigny de Cocody, Abidjan, Côte d’Ivoire; 6 Laboratoire National d’Appui au Développement Agricole (LANADA), Laboratoire Régional de Korhogo, Korhogo, Côte d’Ivoire; 7 The Francis Crick Institute, London, NW1 1AT UK; 8 Department of Infectious Diseases, Imperial College London, London, W12 0NN UK; 9 Wellcome Discovery Research Platforms in Infection, Centre for Infectious Diseases Research in Africa, Institute of Infectious Disease and Molecular Medicine and Department of Medicine, University of Cape Town, Observatory, 7925 Republic of South Africa

**Keywords:** Bacterial profile, Meningitis pathogens, 16S rRNA, Illumina Miseq, Côte d’Ivoire, Abidjan, Korhogo

## Abstract

**Background:**

Bacterial meningitis represents a critical public health concern in sub-Saharan African school settings, where conditions favor its transmission. This study uses 16S rRNA gene sequencing to investigate the presence, relative abundance, and genetic relatedness of the three main bacterial meningitis pathogens in oropharyngeal samples from schoolchildren aged 8–12 years in Côte d’Ivoire.

**Methods:**

76 children from two Ivorian primary schools (37 Korhogo, 39 Abidjan) were followed up for six months. Nucleic acids from oropharyngeal swabs (444 samples) were analyzed by 16S rRNA sequencing, and phylogenetic relationships were inferred using the Maximum Likelihood method.

**Results:**

*H. influenzae* showed the highest carriage prevalence (81.94%, 59/72), Abidjan (81.6%, 31/38) and Korhogo (82.4%, 28/34). *Neisseria meningitidis and Streptococcus pneumoniae* were rare (1.39%, 1/72 and 2.78%, 2/72, respectively) and detected only in Korhogo (2.9% and 5.9%). Genus-level relative abundances were 15.4% (*Haemophilus*), 12.9% (*Streptococcus*) and 5.4% (*Neisseria*). Species-level relative abundances of *Hi*, *Nm*, and *Sp* were 0.16%, 0.03%, and 0.003%, respectively. Mixed-effects logistic regression showed that *Nm* (OR = 0.056, 95% CI: 0.014–0.231, *p* < 0.001) and *Sp* (OR = 0.055, 95% CI: 0.007–0.407, *p* = 0.004) were less likely to colonize than *Hi*. No significant difference was observed between cities (OR = 0.96, 95% CI: 0.58–1.60, *p* = 0.887). High inter-individual variability (SD = 0.91) was observed, with one-third of bacterial species shared between sites.

**Conclusion:**

These findings highlight the utility of 16S rRNA sequencing for culture-independent detection of carriage of key meningitis pathogens, pending further species-level confirmation.

**Supplementary Information:**

The online version contains supplementary material available at https://doi.org/10.1186/s12879-026-13880-9.

## Introduction

Meningitis is an infectious disease involving inflammation of the protective layers of the brain and the spinal cord [1]. While it can be caused by various pathogens, bacterial meningitis is the most dangerous due to its potential to trigger local outbreaks or epidemics. Bacterial meningitis arises when pathogens that colonize the nasopharynx invade the bloodstream, penetrate the blood-brain barrier and reach the subarachnoid space [2].

The most prevalent etiological agents of bacterial meningitis in children and adults are *Streptococcus pneumoniae* [*Sp*], *Neisseria meningitidis* [*Nm*] and *Haemophilus influenzae* serotype b [*Hib*] and are associated with significant morbidity and mortality. In neonates, *Streptococcus agalactiae* [*GBS*] is also one of the main causes of meningitis [3]. These pathogens employ various strategies to survive in the bloodstream and penetrate the central nervous system, such as bacterial encapsulation to resist phagocytosis [4]. Once in the subarachnoid space, they trigger an intense inflammatory response, leading to increased intracranial pressure and cerebral edema [4] .

Delayed diagnosis and treatment of bacterial meningitis can result in irreversible brain damage, making prompt identification and treatment of causative agents essential [5]. Bacterial meningitis causes approximately 125,000 deaths annually in infants and young children, with 96% of these fatalities occurring in developing countries. Mortality rates for affected children can reach 50%, and of those who survive, 25–50% experience long-term neurological complications, such as epilepsy, intellectual disability, or sensorineural hearing loss [6].

Although *Nm*, *Sp* and *Hi* are major causes of meningitis, they are also commonly carried asymptomatically in the oropharynx of healthy individuals. While only a small proportion of carriers develop invasive disease, asymptomatic carriage combined with suboptimal hygiene practices may facilitate pathogen transmission, particularly in crowded settings such as nurseries and schools [7–9].

In urban areas of Côte d’Ivoire, public primary schools have an average of approximately 47 students per classroom [10]. This increased proximity enhances transmission of pathogens, especially those carried in the oro/naso-pharynx. Children not only transmit these pathogens to each other, but can also carry them outside of school, leading to transmission in their homes or communities. Despite advances in immunization programs and prevention strategies, the predominant bacterial agents responsible for causing meningitis including *Sp*, *Nm*, and *Hib* remain a threat to children’s health. 16S rRNA sequencing allows an untargeted survey of bacterial diversity, detecting multiple species simultaneously, including low-abundance or unexpected taxa, and provides phylogenetic and community-level insights that complement targeted PCR-based detection. Previous studies of oropharyngeal samples from schoolchildren aged 8–12 investigated microbiome diversity without examining species level composition [11]. In our earlier studies, we performed targeted PCR for the identification of *Sp* and *Hi* [12] in children presenting with clinical signs of respiratory infection, whereas PCR for *Nm* was carried out only on samples that were positive by culture [11]. While these PCRs provided specific and reliable detection of known pathogens, 16S sequencing offers a broader view of bacterial communities. Therefore, this study aimed to investigate the presence, relative abundance, and genetic relatedness of the three main bacterial meningitis pathogens in oropharyngeal samples from schoolchildren aged 8–12 years in Côte d’Ivoire using the 16S rRNA gene sequencing.

## Methodology

### Study sites and participants

This was a longitudinal study investigating asymptomatic carriage conducted in two public primary schools: one in Korhogo in northern Côte d’Ivoire within the meningitis belt and another in Abidjan in the southern part of the country from November 2020 to April 2021. The study site, characteristics of participants, and sample collection methods have been described previously [11]. All isolates included in this analysis were derived from carriage samples, not from invasive infections.

A list of 8-12-year-old pupils, the age group most susceptible to meningococcal carriage per previous studies in the African meningitis belt, was generated from registration files [13]. Seventy-six (76) pupils (37 in Korhogo and 39 in Abidjan) were randomly selected and enrolled in the study with parental consent and their own assent. Ethical approval was obtained from the national ethical committee (number: IRB000111917) of Côte d’Ivoire prior to sample collection.

### Samples collection and processing

One oropharyngeal swab was collected from each participant during six monthly surveys (S1-S6), conducted at approximately one-month intervals between November 2020 and April 2021. The oropharyngeal samples were collected with a FLOQSwabs^®^ flexible swab with a nylon tip (Copan) using standard methods described elsewhere [11, 14].

### 16S sequencing for bacterial microbiome

DNA was extracted from an aliquot of the swab using the DNeasy PowerSoil kit following the manufacturer’s instructions (Qiagen). To minimize contamination, all extractions were performed in a dedicated clean laboratory space at CSRS, Côte d’Ivoire using sterile consumables. Negative controls (extraction blanks) were included in every batch to monitor potential contamination. DNA concentration was assessed using Qubit DNA quantification kits (HS and BS). The DNA concentrator kit (Zymo) was used to increase the concentration of samples by reducing the elution buffer. Sequencing was performed at WACCBIP, University of Ghana; briefly the V3–V4 hypervariable region of the 16S rRNA gene was amplified using primers 341 F/785R with Illumina adapter overhang sequences [11, 15]. All samples were then sent for library preparation and sequencing on an Illumina MiSeq platform [11].

### 16S rRNA data analysis

Sequencing reads were processed using the DADA2 pipeline (Divisive Amplicon Denoising Algorithm) of R-4.2.1 software [16]. Quality filtering and denoising were performed with the filterAndTrim() function, applying the following parameters: truncLen = c (270,270) for forward and reverse reads, trimLeft = 20, maxN = 0, maxEE = c(5,10), and truncQ = 2. Error rates were modeled using the learnErrors() function on the filtered forward (filtFs) and reverse (filtRs) reads, and the resulting error models were subsequently used for denoising. Dereplication of sequences was carried out using derepFastq (), denoising with dada () and merging paired-end reads with mergePairs(). Chimeric sequences were removed using removeBimeraDenovo() to retain only true biological variants [11].

The resulting amplicon sequence variants (ASVs) were taxonomically classified using the assignTaxonomy() function, with the SILVA database (SILVA_NR_v138) as the reference at 99% sequence similarity [15]. ASVs were aggregated to higher taxonomic levels (phylum, genus, species) for downstream analyses of microbial relative abundances.

To ensure dataset robustness, we applied several filtering steps: taxa with a total abundance ≤ 10 across all samples were removed to reduce noise, samples with zero total abundance were excluded, and only samples with a total abundance ≥ 1000 were retained. Finally, taxa absent from the remaining samples were removed to finalize the dataset.

Negative controls including field controls, blank extraction and library prep were used to monitor potential contamination during sample processing, while positive control samples containing *Escherichia coli* DNA were used to validate the sequencing workflow. Contaminants were defined as ASVs (amplicon sequence variants) detected in blank samples or in negative controls. ASVs present in blanks at levels comparable to or higher than in clinical samples were considered contaminants and removed from downstream analyses to ensure data quality. Contaminants detected in blank samples were addressed using the decontam package, which identifies potential contaminant sequences by comparing prevalence between true samples and blanks [17]. A taxon was considered detected when at least one high-quality read remained after quality filtering, denoising, chimera removal, and contaminant filtering. The cleaned and filtered ASV data were then combined into a phyloseq object for downstream analysis [11].

### Phylogenetic analysis

Twenty representative ASVs were selected from the 6,660 inferred ASVs based on their relative abundance and assignment to the three major bacterial taxa associated with meningitis in this study. For phylogenetic analysis, we focused on the three main bacterial species associated with meningitis detected in our dataset: *Haemophilus influenzae*, *Streptococcus pneumoniae* and *Neisseria meningitidis*. While other bacterial pathogens can cause meningitis, only these three were included in this phylogenetic analysis. Reference genome sequences were retrieved from the PubMLST database for all species, with additional sequences from NCBI for *Haemophilus influenzae* [18, 19]. The selection of reference sequences was guided by stringent quality criteria, including assembly metrics such as the number of contigs and genome size (bp), as well as the recency of the sequences, with preference given to genomes generated within the last 15 years to ensure high-quality references and reliable downstream bioinformatic comparisons.

The V3–V4 regions (nucleotide positions 341–806) were extracted using Unipro UGENE version 53.1 and aligned using MUSCLE [20, 21]. Phylogenetic relationships were inferred using the Maximum Likelihood method under the Kimura 2-parameter substitution model, with 1,000 bootstrap replicates; only bootstrap values ≥ 70% are shown [22].

Initial trees were generated using Neighbor-Joining and BioNJ algorithms based on pairwise distances estimated by the Maximum Composite Likelihood method. All analyses were performed in MEGA12 version 12.1.2 [23] and phylogenetic trees were rooted with the 16S rRNA sequence of *Escherichia coli* K-12 (NR_024570.1) as the outgroup.

Given the short length of the 16S rRNA gene amplicons, the phylogenetic analysis was used for exploratory purposes only, to visualize relationships between representative ASVs and reference sequences, and not as definitive taxonomic confirmation.

### Data and statistical analysis

Data visualization was carried out using the ggplot2 package, and all analyses were performed using R version 4.5.2.

The relative abundances at the genus level were calculated using the *phyloseq* package [24]. The Jaccard similarity index to assess the degree of similarity between the two sites based on the presence or absence of bacteria was calculated using the *vegan* package [25]. Carriage prevalence was calculated as the proportion of positive children among all enrolled participants.

A mixed-effects logistic regression model was fitted to assess factors associated with bacterial colonization. The model included fixed effects for bacterial species (*Species*) and study site (*City*), and a random intercept for each child to account for within-individual correlation due to repeated measurements. The model was implemented using the *glmer* function from the *lme4* package, with a binomial error distribution and logit link.

The model specification was: **Colonized ~ Species + City + (1 | ChildID).**

## Results

A focused analysis was performed on the main bacterial species known to cause meningitis to highlight their prevalence and phylogenetic context.

### Relative abundance of the main bacteria species associated with meningitis

The 16S rRNA sequence data from the 434 samples received after sequencing had an average read length of 287 bases, with 66.74% of reads showing a quality score ≥ 30 and 29.86% showing a score ≥ 20. Prior to quality filtering, the median read depth per sample was 124,656 reads, compared with 90,402 reads in the controls (Supplementary Fig. 1), indicating a higher sequencing signal in biological samples than in negative controls. From 36.556.031 raw reads, sequential filtering, denoising, merging, and chimera removal refined the dataset to 18.953.527 high-quality reads (51,85%).

**Fig. 1:**
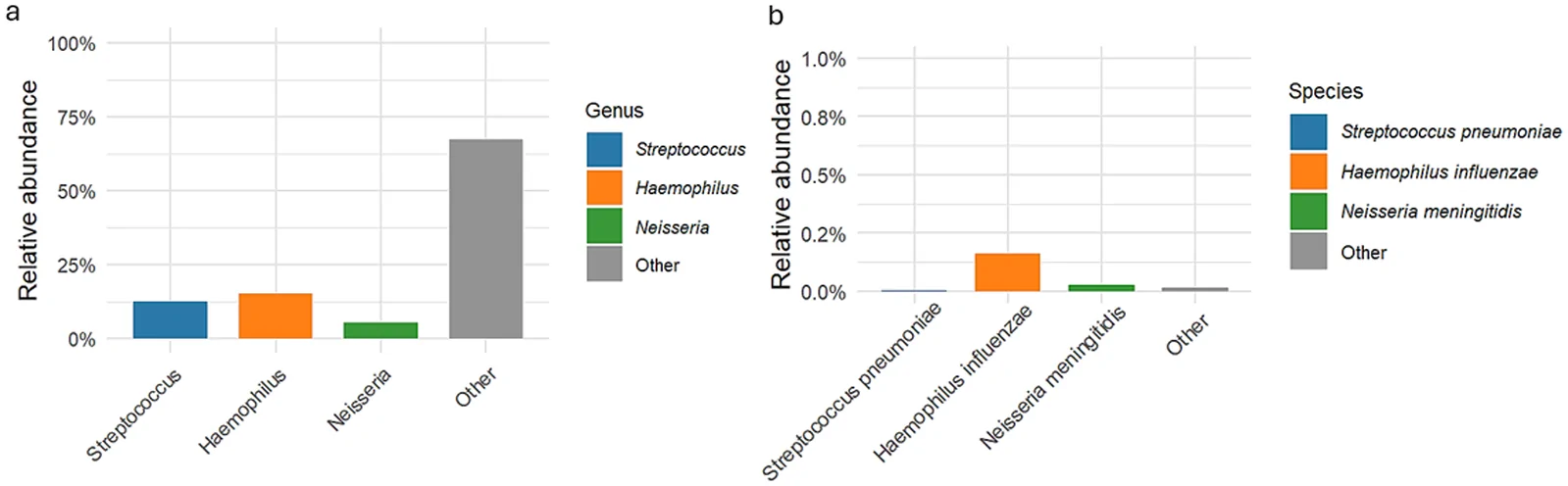
Bar plot showing the main bacteria associated with meningitis based on relative abundance at the genus (**a**) and species (**b**) levels. The “Other” category in the bar plot represents bacterial genera or species that are detected in the samples but individually have low relative abundance

The analyses identified 312 genera and 199 species. Among these, the genera *Haemophilus*,* Streptococcus*, and *Neisseria* were identified with relative abundances of 15.4%, 12.9%, and 5.4%, respectively. The remaining 66.3% of sequences were distributed across a wide range of other genera (Fig. 1a).

At the species level, *Hi* accounted for 0.16% of total sequences, *Nm* for 0.03%, *Sp* for 0.003%; species individually representing less than 0.01% of total reads were grouped under the category “Other”, which collectively accounted for 0.015% of the total relative abundance (Fig. 1b). The other bacteria that were identified comprise a diverse consortium of facultative and obligate anaerobes (Supplementary Table 1).

### Distribution of bacterial species across study sites

**Fig. 2:**
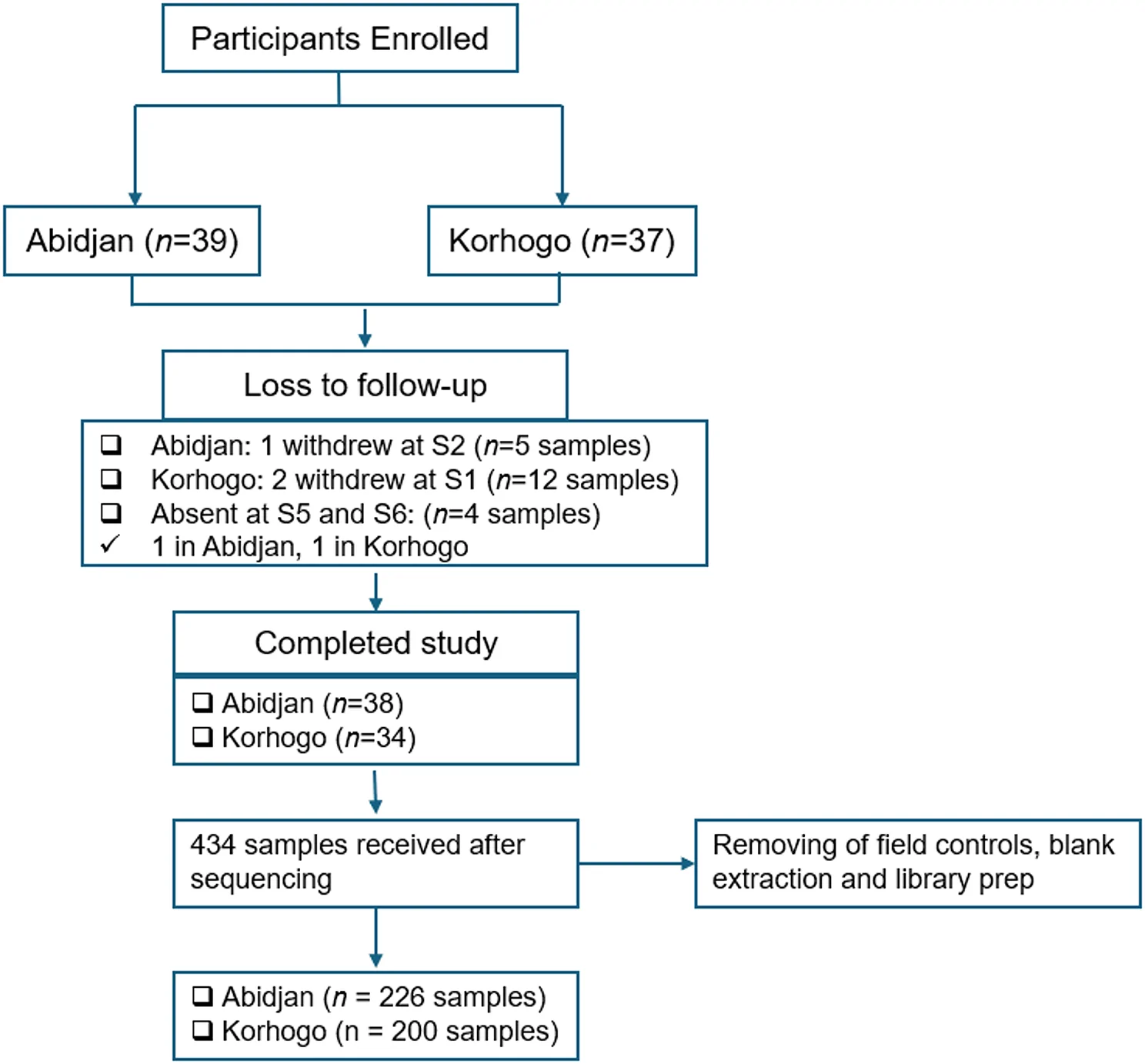
Flow diagram of participant enrollment and monthly follow-up among children in Abidjan and Korhogo, Côte d’Ivoire during the study period

Primary school children in Korhogo (*n* = 37) and Abidjan (*n* = 39) were followed up for six months with monthly oropharyngeal sampling. Of 434 samples received after sequencing, 426 passed the filter for 16S rRNA analysis (Abidjan: *n* = 226 samples; Korhogo: *n* = 200 samples) (Fig. 2).

The cohort was predominantly female, comprising 49 (64.5%) girls and 27 (35.5%) boys overall. In contrast, the gender distribution in Korhogo was more balanced, with 19 (51.4%) girls and 18 (48.6%) boys. All participants were within the required age range of 8–12 years, with most children belonging to the 9- and 10-year-old age groups at both sites (Table 1).

**Table 1:** Demographic characteristics of the participants enrolled in this study between November 2020 and April 2021

Gender	Abidjan	%	Korhogo	%
Girls	30	76.9	19	51.4
Boys	9	23.1	18	48.6
Total	39		37	
**Age (years)**				
8	5	12.8	6	16.2
9	10	25.6	18	48.6
10	12	30.8	10	27
11	8	20.5	3	8.1
12	4	10.3	0	0
Total	39		37	

Overall, *Hi* showed the highest carriage prevalence at 81.94% (59/72), whereas *Nm* and *Sp* were detected at much lower prevalences of 1.39% (1/72) and 2.78% (2/72), respectively. *Hi* was the most frequently detected species in both cities. In Abidjan, 31 out of 38 children were colonized, corresponding to a prevalence of 81.6%. Similarly, in Korhogo, 28 out of 34 children were colonized, representing a prevalence of 82.4%. In contrast, no colonization by *Nm* and *Sp* was detected among children in Abidjan. In Korhogo, *Nm* was detected in 1 out of 34 children (2.9%), while *Sp* was identified in 2 out of 34 children (5.9%) (Table 2). Among the colonized children, one individual from Korhogo tested positive for the Sp at both visit 2 and visit 3. After accounting for repeated sampling within children using a mixed-effects logistic regression, the probability of colonization differed significantly among bacterial species. *Nm* (OR = 0.056, 95% CI: 0.014–0.231, *p* < 0.001) and *Sp* (OR = 0.055, 95% CI: 0.007–0.407, *p* = 0.004) were less likely to colonize than *Hi*. No significant difference was observed between cities (OR = 0.96, 95% CI: 0.58–1.60, *p* = 0.887). The standard deviation of the random intercept by child was 0.91, indicating substantial inter-individual variability.

The Jaccard index indicated that approximately one-third (33.3%) of bacterial species were shared between the two sites, reflecting moderate similarity in species composition.

**Table 2:** Prevalence in the two study sites in Cote d’Ivoire of meningitis bacteria among children aged 8–12 years between November 2020 and April 2021

City	Species	*N*° times positive	*N*° children	Proportion %
Abidjan *n* = 38	*Haemophilus influenzae*	1	9	29
		2	7	22.6
		3	5	16.1
		4	4	12.9
		5	5	16.1
		6	1	3.23
		Total	31/38	81.6
Korhogo *n* = 34	*Haemophilus influenzae*	1	9	32.1
		2	6	21.4
		3	5	17.9
		4	3	10.7
		5	3	10.7
		6	2	7.14
		Total	28/34	82.4
	*Neisseria meningitidis*	1	1	2.94
	*Streptococcus pneumoniae*	1	2	5.9

**Fig. 3:**
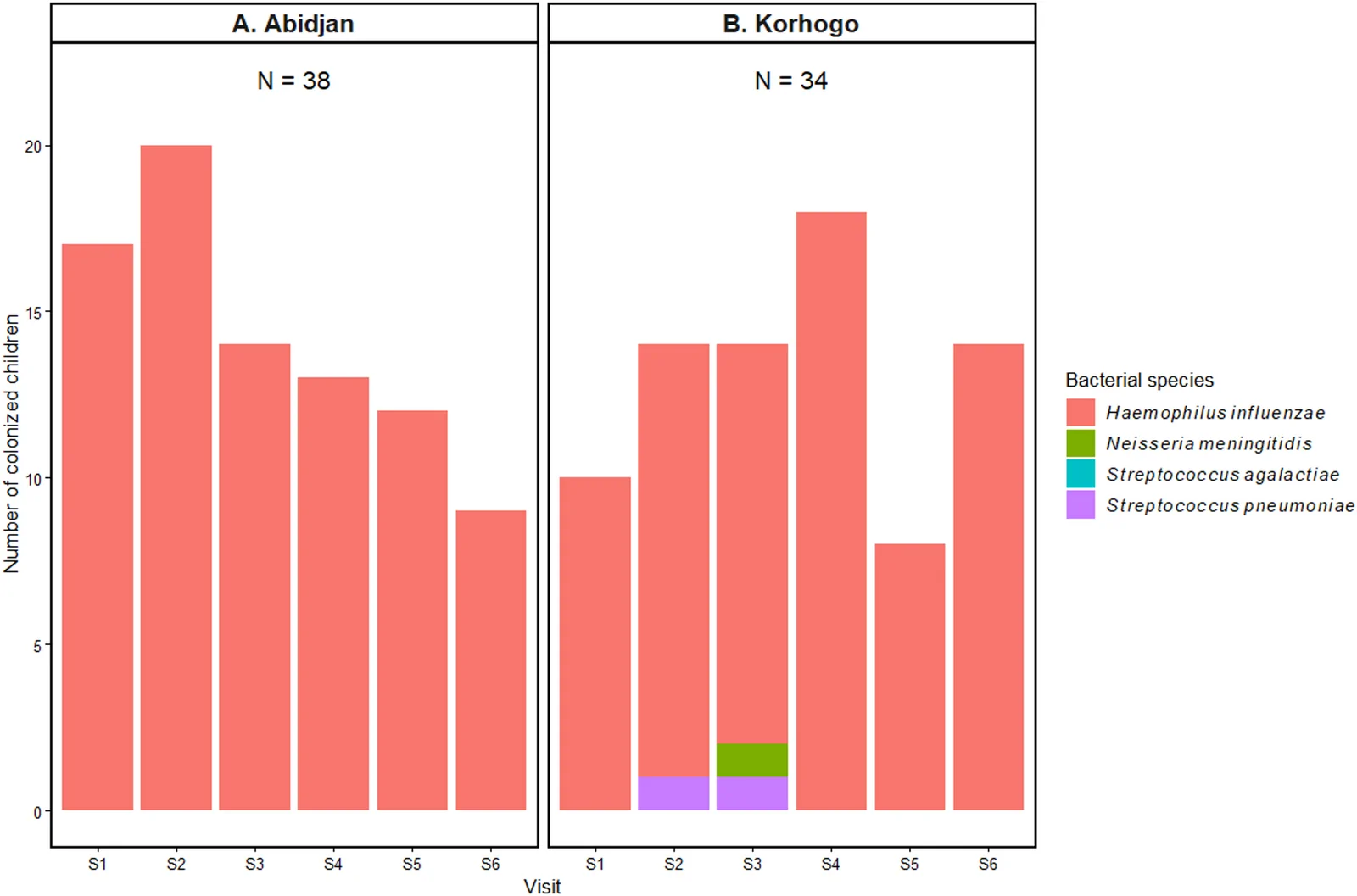
Bar charts showing the number of colonized children by three meningitis-associated bacteria (*Streptococcus pneumoniae*, *Neisseria meningitidis*, *Haemophilus influenzae*,* Streptococcus agalactiae*) by visit and site (A: Abidjan, B: Korhogo). Visits are labeled with ‘S’ to indicate surveys (e.g., S1 = Survey 1)

Oropharyngeal carriage of meningitis-associated bacteria varied across visits (S1 to S6) and study sites (Abidjan and Korhogo). *Hi* was present on all visits, *Nm* was detected only at visit 3 by 16S rRNA at the Korhogo site. *Sp* was observed at visits 3 and 4 at the Korhogo site but was absent in Abidjan using the 16S RNA gene sequencing (Fig. 3).

### Phylogenetic analysis of meningitis bacteria

Based on the 6,660 Amplicon Sequence Variant (ASV) generated, *Hi* was associated with 17 ASV (0.26%), *Nm* with 1 ASV (0.02%), *SP* with 2 ASV (0.03%). The ASVs corresponding to the study sequences are presented in Supplementary Table 2.

**Fig. 4:**
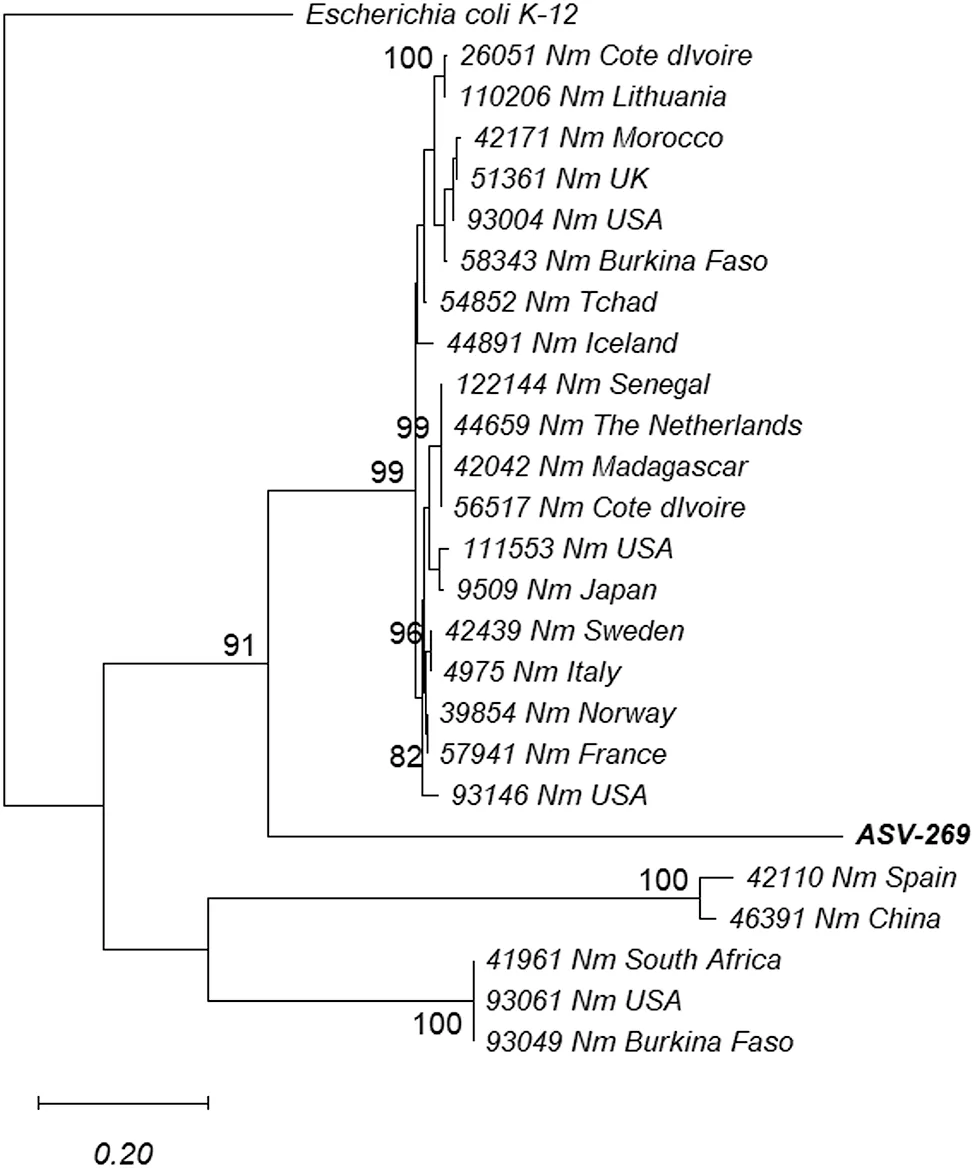
Maximum-likelihood molecular phylogenetic analysis of *Neisseria meningitidis* Amplicon Sequence Variant based on 16S rRNA gene sequences. Sequences generated in this study are shown in bold. The tree was rooted using *Escherichia coli* K-12 (NR_024570.1) as outgroup, and bootstrap values (> 70%) are indicated at the branch nodes

Phylogenetic analysis of 16S rRNA sequences from *Neisseria meningitidis*, including ASV-269, showed genetic similarity among the sequences. ASV-269 clustered closely with the *N. meningitidis* strains included in the study, indicating a close genetic relationship (Fig. 4 and Supplementary Fig. 2).

**Fig. 5:**
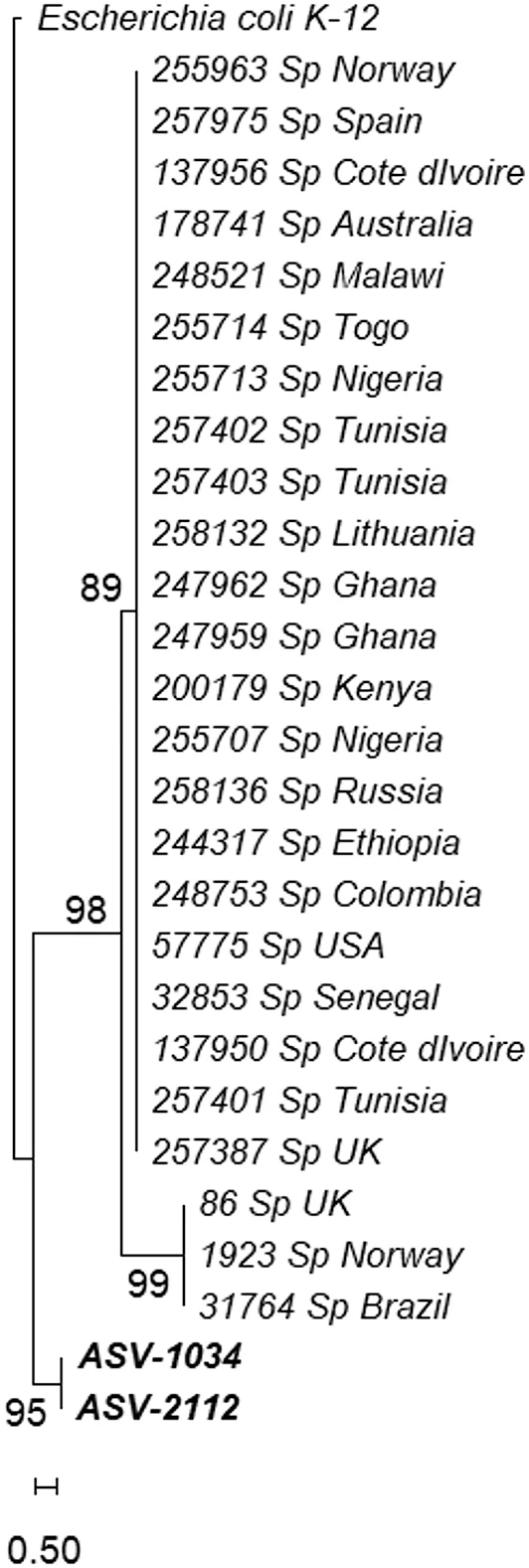
Maximum-likelihood molecular phylogenetic analysis of *Streptococcus pneumoniae* Amplicon Sequence Variant based on 16S rRNA gene sequences. Sequences generated in this study are shown in bold. The tree was rooted using *Escherichia coli* K-12 (NR_024570.1) as outgroup, and bootstrap values (> 70%) are indicated at the branch nodes

The phylogenetic analysis of 16S rRNA sequences from *Streptococcus* species, including ASV-2112 and ASV-1034 variants of *S. pneumoniae*, showed that the two ASV clustered closely together, indicating high genetic similarity. These results highlight the close relationship of the ASVs with the reference strains included in the analysis (Fig. 5).

**Fig. 6:**
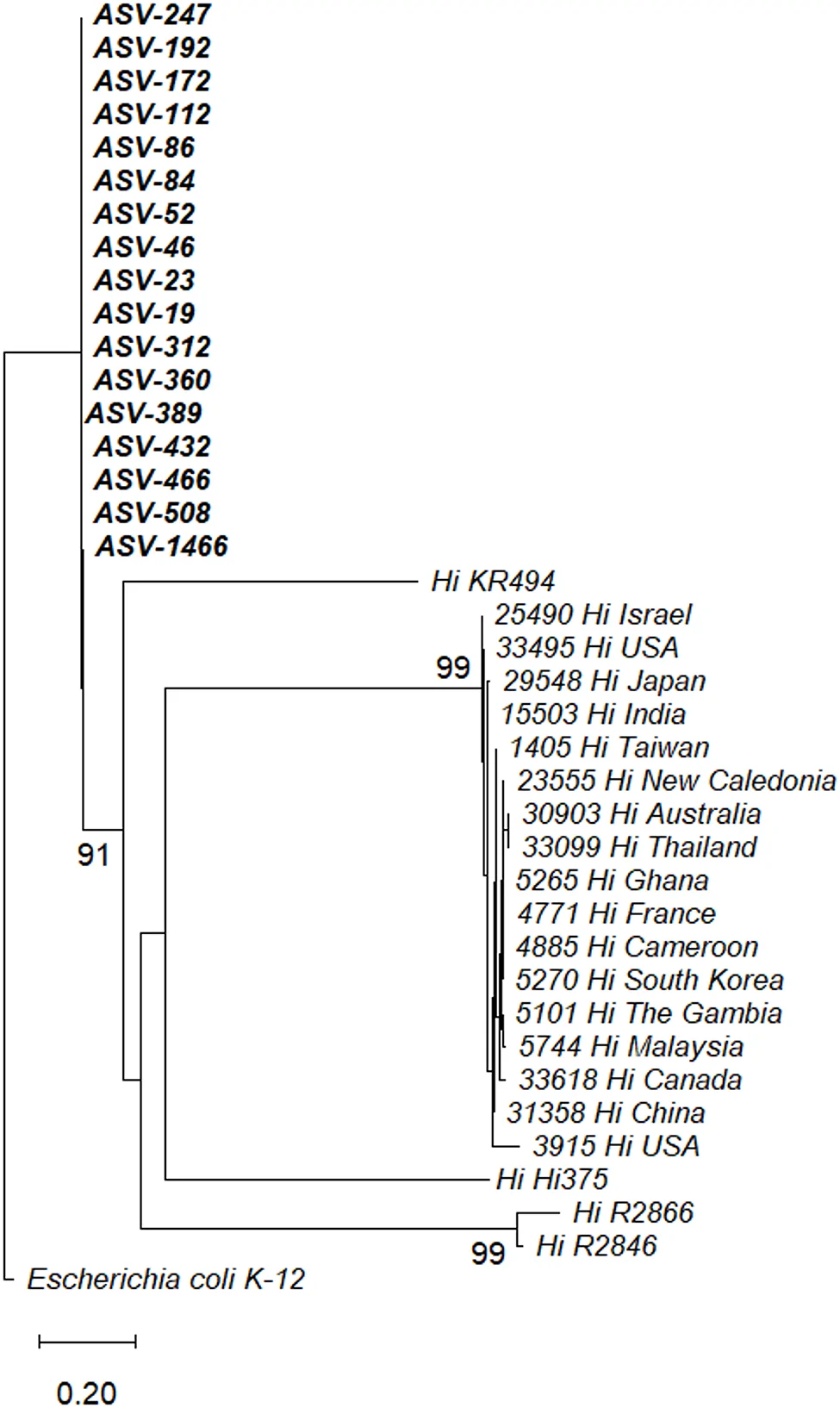
Maximum-likelihood molecular phylogenetic analysis of *Haemophilus influenzae* Amplicon Sequence Variant based on 16S rRNA gene sequences. Sequences generated in this study are shown in bold. The tree was rooted using *Escherichia coli* K-12 (NR_024570.1) as outgroup, and bootstrap values (> 70%) are indicated at the branch nodes

The phylogenetic analysis of 16S rRNA sequences from *Haemophilus influenzae*, including multiple ASVs (ASV-1466, ASV-23, ASV-19, etc.) and reference strains, showed that these sequences clustered closely together with strong bootstrap support, indicating genetic similarity (Fig. 6).

## Discussion

This study provides a culture-independent overview of meningitis-associated bacteria carried by schoolchildren in two Ivorian settings using 16S rRNA sequencing. We observed that *Hi* showed the highest carriage prevalence at 81.94% (59/72), whereas *Nm* and *Sp* were detected at much lower prevalences of 1.39% (1/72) and 2.78% (2/72), respectively. *Hi* was frequently detected in both Abidjan and Korhogo (31/38, 81.6% and 28/34, 82.4%, respectively), whereas *Nm* and *Sp* were rarely detected. Differences in microbial composition were observed between Abidjan and Korhogo, with limited species overlap. Colonization probability varied significantly by species, with *Nm* and *Sp* were less likely to colonize than *Hi*. No significant difference was found between cities, whereas substantial inter-individual variability was observed. These findings frame *Hi* as a frequent but low-abundance component of the oropharyngeal microbiota and *Nm*/*Sp* as infrequent colonizers in this cohort. Its presence across all six sampling visits suggests that *Hi* is a stable component of the respiratory microbiota, While colonization is often asymptomatic, invasive disease is mainly caused by typeable strains, particularly type b, which are responsible for meningitis and sepsis. In contrast, non-typeable strains, which are the predominant colonizing forms, rarely cause invasive infections but may do so under specific conditions such as trauma or disruption of mucosal barriers [26]. Although, *Hi* were detected in many samples (high prevalence), while their relative abundance within individual samples remained very low. Our observations are consistent with the literature positioning non-typeable *Hi* can asymptomatically colonize the oropharynx or nasopharynx of healthy individuals, including children and adults, supporting its role as an opportunistic commensal organism [27, 28].

The prevalence of *Nm* observed in this study (1.39%) is consistent with low carriage rates reported in African settings. Large multicountry studies conducted in the African meningitis belt using nasopharyngeal or combined sampling strategies have reported prevalence estimates ranging from approximately 2% to 4%, including 3.4% in a seven-country carriage study and 3.6% in the MenAfriCar consortium [29, 30]. *Nm* was detected in only one sample from Korhogo, a city located within the African meningitis belt. Nevertheless, *Nm* carriage and meningococcal outbreaks are known to occur more frequently during the dry season in this region, when environmental conditions such as dust, low humidity, and increased close contact among individuals may facilitate transmission [31]. Its presence only on visit 3 (December) suggests potential seasonality, consistent with other findings in West Africa [13, 32, 33].

For *Sp*, reported carriage rates in children vary widely from approximately 26.7% to 90.7% [34–36]. Although nasopharyngeal sampling remains the reference method for pneumococcal carriage studies, oropharyngeal sampling has also been used in some African studies, particularly in adults. A Kenyan study conducted in 2020 reported that oropharyngeal swabs identified approximately 5.7% of additional pneumococcal carriers beyond those detected by nasopharyngeal sampling [37]. Therefore, the comparatively low prevalence observed in our study (2.78%) may partly reflect methodological differences related to the use of oropharyngeal swabs, as well as differences in vaccination status [38, 39]. In addition, the introduction of PCV13 into the Expanded Programme on Immunization in Côte d’Ivoire in 2013, together with high vaccine coverage estimated at approximately 99% in 2017, may also have contributed to reduced *Sp* carriage [36]. Its sporadic presence at visits 3 (December) and 4 (January) suggests temporal variation, which may correspond to seasonal transmission patterns as observed in sub-Saharan settings [38].

In contrast, although *Group B Streptococcus* (GBS) is an important pathogen in neonates and pregnant women, it is less commonly detected in oropharyngeal samples because it predominantly colonizes the gastrointestinal and genitourinary tracts [39].Therefore, its absence in the present study may be related to the sampling site and the characteristics of the study population rather than a true epidemiological absence. Overall, these findings suggest that healthy schoolchildren harbor a diverse oropharyngeal microbiota in which potentially pathogenic bacterial species may be present at low abundance, consistent with asymptomatic carriage reported in previous studies [40, 41].

The high abundance of “other” taxa reflects the complexity of the oropharyngeal microbiota, which is largely composed of commensal organisms that may influence pathogen colonization dynamics. Although these taxa are not classical meningitis pathogens, some opportunistic species, such as *Staphylococcus aureus* and *Acinetobacter lwoffii*, have been reported in invasive infections, particularly in immunocompromised or hospitalized patients [42, 43]. Dysbiosis of the oropharyngeal microbiome may contribute to the shift from asymptomatic carriage to invasive disease [12, 44, 45]. This potential underscores the importance of continuous surveillance, particularly in school settings where close contact may facilitate transmission [46, 47].

The Jaccard index showed 33.33% similarity between the two study sites, suggesting moderate differences in species composition. These variations may be influenced by environmental and lifestyle factors, including antibiotic use, diet, urbanization, and climate, as previously reported in similar settings [48, 49].

Species-level identification based solely on 16S rRNA (V3–V4 region) cannot reliably resolve serotypes or clonal complexes, which limits inferences about hyperinvasive lineages and vaccine-relevant serogroups, despite the advantage of allowing simultaneous detection of multiple pathogens in a single assay. For this reason, phylogenetic interpretations should be made cautiously and cannot infer circulation of specific lineages.

Compared with conventional culture, 16S rRNA gene sequencing provides a culture-independent, broad-range approach for bacterial detection, enabling the identification of fastidious and uncultivable organisms as well as comprehensive profiling of microbial communities [50, 51]. Recent comparative studies and systematic reviews have shown that 16S rRNA gene sequencing is most valuable as a complementary diagnostic tool, particularly in cases where conventional microbiological methods yield negative or inconclusive results [50, 51].

Our work was conducted in the context of a carriage study, but in clinical practice, PCR is preferred for routine diagnosis when a specific pathogen is suspected, due to its high sensitivity and rapid turnaround time. In contrast, 16S rRNA gene sequencing can detect a wide range of bacteria simultaneously and is particularly useful in culture-negative or diagnostically challenging cases, or when a broader characterization of bacterial diversity is required [50–52]. Therefore, the choice of diagnostic strategies should be guided by clinical presentation, urgency, and available laboratory resources.

Future studies integrating high-resolution genomic methods with epidemiological and clinical data would improve understanding of strains with invasive potential and support targeted public health strategies [53].

## Conclusion

Our study identified a low prevalence of carriage of *Haemophilus influenzae*, *Neisseria meningitidis*, and *Streptococcus pneumoniae* in oropharyngeal samples from healthy schoolchildren in Côte d’Ivoire, suggesting limited carriage of these meningitis-associated bacteria in this population. In addition, the type of *H. influenzae* detected could not be determined, limiting interpretation of its invasive potential. Overall, these findings reflect asymptomatic colonization rather than evidence of active meningitis disease transmission. They also highlight substantial inter-individual variability and the combined influence of methodological and epidemiological factors. Furthermore, opportunistic bacterial species were detected, underscoring the diversity of the oropharyngeal microbiota in healthy children. Although not primary meningitis pathogens, these organisms may act as opportunistic pathogens under specific conditions. Finally, 16S rRNA gene sequencing provided valuable insights into bacterial diversity but has limited resolution at the strain level, highlighting the need for complementary high-resolution genomic approaches for future surveillance.

## Supplementary Information

Below is the link to the electronic supplementary material.

Supplementary Material 1

## Data Availability

The 16S rRNA datasets presented in this study can be found in online repositories. The names of the repository/repositories and accession number(s) can be found below: [PRJNA1064912 - SRA - NCBI] (https://www.ncbi.nlm.nih.gov/sra/PRJNA1064912), from SRX23222127 to SRX23221695.

## Abbreviations

ASV: Amplicon sequence variant
CNESVS: Comité national d’éthique des sciences de la vie et de la santé [National Ethics Committee for Life and Health Sciences
CNS: Central Nervous System
CSRS: Centre Suisse de Recherches Scientifiques en Côte d’Ivoire
DNA: Deoxyribonucleic acid
GBS: Group B Streptococcus
Hi: Haemophilus influenzae
LANADA: Laboratoire National d’Appui au Développement Agricole
Nm: Neisseria meningitidis
N°: Number
PCR: Polymerase Chain Reaction
PNSSU: Programme National de la Santé Scolaire et Universitaire [National school and university health program
RNA: Ribonucleic acid
Sp: Streptococcus pneumoniae
WACCBIP: West African Centre for Cell Biology of Infectious Pathogens
