# Supplementary material for: Profile of bacterial meningitis pathogens circulating in a cohort of school children in Côte d’Ivoire using 16S rRNA gene sequencing
Source: BMC Infect Dis. 2026 Jun 30;26:1684. doi: 10.1186/s12879-026-13880-9 (PMC13591789; doi:10.1186/s12879-026-13880-9)
Supplement: Supplementary file 1 [file 12879_2026_13880_MOESM1_ESM.docx]

**Supplementary Table 1:** List of other species identified in the study using 16S rRNA sequencing

| **Kingdom** | **Phylum** | **Class** | **Order** | **Family** | **Genus** | **Species** |
| --- | --- | --- | --- | --- | --- | --- |
| Bacteria | Firmicutes | Bacilli | Lactobacillales | Aerococcaceae | Abiotrophia | *Abiotrophia defectiva* |
| Bacteria | Proteobacteria | Gammaproteobacteria | Pseudomonadales | Moraxellaceae | Acinetobacter | *Acinetobacter lwoffii* |
| Bacteria | Proteobacteria | Gammaproteobacteria | Pseudomonadales | Moraxellaceae | Acinetobacter | *Acinetobacter radioresistens* |
| Bacteria | Proteobacteria | Gammaproteobacteria | Pseudomonadales | Moraxellaceae | Acinetobacter | *Acinetobacter soli* |
| Bacteria | Proteobacteria | Gammaproteobacteria | Pseudomonadales | Moraxellaceae | Acinetobacter | *Acinetobacter ursingii* |
| Bacteria | Proteobacteria | Gammaproteobacteria | Pasteurellales | Pasteurellaceae | Actinobacillus | *Actinobacillus ureae* |
| Bacteria | Actinobacteriota | Actinobacteria | Actinomycetales | Actinomycetaceae | Actinomyces | *Actinomyces graevenitzii* |
| Bacteria | Actinobacteriota | Actinobacteria | Actinomycetales | Actinomycetaceae | Actinomyces | *Actinomyces naeslundii* |
| Bacteria | Actinobacteriota | Actinobacteria | Actinomycetales | Actinomycetaceae | Actinomyces | *Actinomyces odontolyticus* |
| Bacteria | Actinobacteriota | Actinobacteria | Propionibacteriales | Nocardioidaceae | Aeromicrobium | *Aeromicrobium halocynthiae* |
| Bacteria | Proteobacteria | Gammaproteobacteria | Aeromonadales | Aeromonadaceae | Aeromonas | *Aeromonas caviae* |
| Bacteria | Proteobacteria | Gammaproteobacteria | Pasteurellales | Pasteurellaceae | Aggregatibacter | *Aggregatibacter actinomycetemcomitans* |
| Bacteria | Proteobacteria | Gammaproteobacteria | Pasteurellales | Pasteurellaceae | Aggregatibacter | *Aggregatibacter aphrophilus* |
| Bacteria | Proteobacteria | Gammaproteobacteria | Pasteurellales | Pasteurellaceae | Aggregatibacter | *Aggregatibacter segnis* |
| Bacteria | Bacteroidota | Bacteroidia | Bacteroidales | Prevotellaceae | Alloprevotella | *Alloprevotella rava* |
| Bacteria | Bacteroidota | Bacteroidia | Bacteroidales | Prevotellaceae | Alloprevotella | *Alloprevotella tannerae* |
| Bacteria | Campilobacterota | Campylobacteria | Campylobacterales | Arcobacteraceae | Arcobacter | *Arcobacter butzleri* |
| Bacteria | Campilobacterota | Campylobacteria | Campylobacterales | Arcobacteraceae | Arcobacter | *Arcobacter lanthieri* |
| Bacteria | Actinobacteriota | Coriobacteriia | Coriobacteriales | Atopobiaceae | Atopobium | *Atopobium parvulum* |
| Bacteria | Bacteroidota | Bacteroidia | Bacteroidales | Bacteroidaceae | Bacteroides | *Bacteroides graminisolvens* |
| Bacteria | Actinobacteriota | Actinobacteria | Frankiales | Geodermatophilaceae | Blastococcus | *Blastococcus aggregatus* |
| Bacteria | Actinobacteriota | Actinobacteria | Micrococcales | Brevibacteriaceae | Brevibacterium | *Brevibacterium pityocampae* |
| Bacteria | Proteobacteria | Alphaproteobacteria | Caulobacterales | Caulobacteraceae | Brevundimonas | *Brevundimonas faecalis* |
| Bacteria | Proteobacteria | Alphaproteobacteria | Caulobacterales | Caulobacteraceae | Brevundimonas | *Brevundimonas terrae* |
| Bacteria | Firmicutes | Bacilli | Erysipelotrichales | Erysipelotrichaceae | Bulleidia | *Bulleidia extructa* |
| Bacteria | Campilobacterota | Campylobacteria | Campylobacterales | Campylobacteraceae | Campylobacter | *Campylobacter concisus* |
| Bacteria | Campilobacterota | Campylobacteria | Campylobacterales | Campylobacteraceae | Campylobacter | *Campylobacter gracilis* |
| Bacteria | Campilobacterota | Campylobacteria | Campylobacterales | Campylobacteraceae | Campylobacter | *Campylobacter showae* |
| Bacteria | Bacteroidota | Bacteroidia | Flavobacteriales | Flavobacteriaceae | Capnocytophaga | *Capnocytophaga gingivalis* |
| Bacteria | Bacteroidota | Bacteroidia | Flavobacteriales | Flavobacteriaceae | Capnocytophaga | *Capnocytophaga granulosa* |
| Bacteria | Bacteroidota | Bacteroidia | Flavobacteriales | Flavobacteriaceae | Capnocytophaga | *Capnocytophaga leadbetteri* |
| Bacteria | Bacteroidota | Bacteroidia | Flavobacteriales | Flavobacteriaceae | Capnocytophaga | *Capnocytophaga sputigena* |
| Bacteria | Proteobacteria | Gammaproteobacteria | Cardiobacteriales | Cardiobacteriaceae | Cardiobacterium | *Cardiobacterium hominis* |
| Bacteria | Firmicutes | Clostridia | Lachnospirales | Lachnospiraceae | Catonella | *Catonella morbi* |
| Bacteria | Proteobacteria | Gammaproteobacteria | Cellvibrionales | Cellvibrionaceae | Cellvibrio | *Cellvibrio mixtus* |
| Bacteria | Verrucomicrobiota | Chlamydiae | Chlamydiales | Chlamydiaceae | Chlamydia | *Chlamydia pneumoniae* |
| Bacteria | Bacteroidota | Bacteroidia | Flavobacteriales | Weeksellaceae | Chryseobacterium | *Chryseobacterium hominis* |
| Bacteria | Firmicutes | Bacilli | Bacillales | Planococcaceae | Chryseomicrobium | *Chryseomicrobium imtechense* |
| Bacteria | Bacteroidota | Bacteroidia | Flavobacteriales | Weeksellaceae | Cloacibacterium | *Cloacibacterium normanense* |
| Bacteria | Actinobacteriota | Actinobacteria | Corynebacteriales | Corynebacteriaceae | Corynebacterium | *Corynebacterium appendicis* |
| Bacteria | Actinobacteriota | Actinobacteria | Corynebacteriales | Corynebacteriaceae | Corynebacterium | *Corynebacterium argentoratense* |
| Bacteria | Actinobacteriota | Actinobacteria | Corynebacteriales | Corynebacteriaceae | Corynebacterium | *Corynebacterium durum* |
| Bacteria | Actinobacteriota | Actinobacteria | Corynebacteriales | Corynebacteriaceae | Corynebacterium | *Corynebacterium matruchotii* |
| Bacteria | Actinobacteriota | Actinobacteria | Corynebacteriales | Corynebacteriaceae | Corynebacterium | *Corynebacterium provencense* |
| Bacteria | Actinobacteriota | Actinobacteria | Propionibacteriales | Propionibacteriaceae | Cutibacterium | *Cutibacterium acnes* |
| Bacteria | Actinobacteriota | Actinobacteria | Propionibacteriales | Propionibacteriaceae | Cutibacterium | *Cutibacterium granulosum* |
| Bacteria | Actinobacteriota | Actinobacteria | Propionibacteriales | Propionibacteriaceae | Cutibacterium | *Cutibacterium namnetense* |
| Bacteria | Deinococcota | Deinococci | Deinococcales | Deinococcaceae | Deinococcus | *Deinococcus geothermalis* |
| Bacteria | Actinobacteriota | Actinobacteria | Micrococcales | Demequinaceae | Demequina | *Demequina globuliformis* |
| Bacteria | Firmicutes | Bacilli | Lactobacillales | Carnobacteriaceae | Desemzia | *Desemzia incerta* |
| Bacteria | Proteobacteria | Alphaproteobacteria | Rhizobiales | Devosiaceae | Devosia | *Devosia chinhatensis* |
| Bacteria | Firmicutes | Negativicutes | Veillonellales-Selenomonadales | Veillonellaceae | Dialister | *Dialister invisus* |
| Bacteria | Actinobacteriota | Actinobacteria | Corynebacteriales | Dietziaceae | Dietzia | *Dietzia natronolimnaea* |
| Bacteria | Actinobacteriota | Actinobacteria | Corynebacteriales | Dietziaceae | Dietzia | *Dietzia timorensis* |
| Bacteria | Firmicutes | Bacilli | Lactobacillales | Carnobacteriaceae | Dolosigranulum | *Dolosigranulum pigrum* |
| Bacteria | Bacteroidota | Bacteroidia | Bacteroidales | Dysgonomonadaceae | Dysgonomonas | *Dysgonomonas oryzarvi* |
| Bacteria | Proteobacteria | Gammaproteobacteria | Burkholderiales | Neisseriaceae | Eikenella | *Eikenella corrodens* |
| Bacteria | Bacteroidota | Bacteroidia | Flavobacteriales | Weeksellaceae | Empedobacter | *Empedobacter brevis* |
| Bacteria | Bacteroidota | Bacteroidia | Flavobacteriales | Weeksellaceae | Empedobacter | *Empedobacter falsenii* |
| Bacteria | Proteobacteria | Gammaproteobacteria | Pseudomonadales | Moraxellaceae | Faucicola | *Faucicola mancuniensis* |
| Bacteria | Firmicutes | Clostridia | Peptostreptococcales-Tissierellales | Peptostreptococcaceae | Filifactor | *Filifactor alocis* |
| Bacteria | Bacteroidota | Bacteroidia | Chitinophagales | Chitinophagaceae | Flavisolibacter | *Flavisolibacter tropicus* |
| Bacteria | Bacteroidota | Bacteroidia | Flavobacteriales | Flavobacteriaceae | Flavobacterium | *Flavobacterium succinicans* |
| Bacteria | Fusobacteriota | Fusobacteriia | Fusobacteriales | Fusobacteriaceae | Fusobacterium | *Fusobacterium massiliense* |
| Bacteria | Fusobacteriota | Fusobacteriia | Fusobacteriales | Fusobacteriaceae | Fusobacterium | *Fusobacterium necrophorum* |
| Bacteria | Fusobacteriota | Fusobacteriia | Fusobacteriales | Fusobacteriaceae | Fusobacterium | *Fusobacterium nucleatum* |
| Bacteria | Fusobacteriota | Fusobacteriia | Fusobacteriales | Fusobacteriaceae | Fusobacterium | *Fusobacterium periodonticum* |
| Bacteria | Firmicutes | Bacilli | Staphylococcales | Gemellaceae | Gemella | *Gemella massiliensis* |
| Bacteria | Firmicutes | Bacilli | Staphylococcales | Gemellaceae | Gemella | *Gemella morbillorum* |
| Bacteria | Firmicutes | Bacilli | Staphylococcales | Gemellaceae | Gemella | *Gemella parahaemolysans* |
| Bacteria | Firmicutes | Bacilli | Staphylococcales | Gemellaceae | Gemella | *Gemella sanguinis* |
| Bacteria | Firmicutes | Bacilli | Lactobacillales | Carnobacteriaceae | Granulicatella | *Granulicatella elegans* |
| Bacteria | Proteobacteria | Gammaproteobacteria | Pasteurellales | Pasteurellaceae | Haemophilus | *Haemophilus haemolyticus* |
| Bacteria | Proteobacteria | Gammaproteobacteria | Pasteurellales | Pasteurellaceae | Haemophilus | *Haemophilus parainfluenzae* |
| Bacteria | Proteobacteria | Gammaproteobacteria | Pasteurellales | Pasteurellaceae | Haemophilus | *Haemophilus pittmaniae* |
| Bacteria | Proteobacteria | Gammaproteobacteria | Pasteurellales | Pasteurellaceae | Haemophilus | *Haemophilus sputorum* |
| Bacteria | Bacteroidota | Bacteroidia | Chitinophagales | Chitinophagaceae | Hydrotalea | *Hydrotalea flava* |
| Bacteria | Proteobacteria | Gammaproteobacteria | Burkholderiales | Neisseriaceae | Kingella | *Kingella oralis* |
| Bacteria | Actinobacteriota | Actinobacteria | Micrococcales | Intrasporangiaceae | Knoellia | *Knoellia flava* |
| Bacteria | Actinobacteriota | Actinobacteria | Micrococcales | Dermacoccaceae | Kytococcus | *Kytococcus sedentarius* |
| Bacteria | Firmicutes | Clostridia | Lachnospirales | Lachnospiraceae | Lachnoanaerobaculum | *Lachnoanaerobaculum umeaense* |
| Bacteria | Firmicutes | Bacilli | Lactobacillales | Lactobacillaceae | Lactobacillus | *Lactobacillus agilis* |
| Bacteria | Firmicutes | Bacilli | Lactobacillales | Lactobacillaceae | Lactobacillus | *Lactobacillus delbrueckii* |
| Bacteria | Firmicutes | Bacilli | Lactobacillales | Lactobacillaceae | Lactobacillus | *Lactobacillus frumenti* |
| Bacteria | Firmicutes | Bacilli | Lactobacillales | Lactobacillaceae | Lactobacillus | *Lactobacillus kunkeei* |
| Bacteria | Proteobacteria | Gammaproteobacteria | Burkholderiales | Burkholderiaceae | Lautropia | *Lautropia mirabilis* |
| Bacteria | Fusobacteriota | Fusobacteriia | Fusobacteriales | Leptotrichiaceae | Leptotrichia | *Leptotrichia buccalis* |
| Bacteria | Fusobacteriota | Fusobacteriia | Fusobacteriales | Leptotrichiaceae | Leptotrichia | *Leptotrichia goodfellowii* |
| Bacteria | Fusobacteriota | Fusobacteriia | Fusobacteriales | Leptotrichiaceae | Leptotrichia | *Leptotrichia hofstadii* |
| Bacteria | Fusobacteriota | Fusobacteriia | Fusobacteriales | Leptotrichiaceae | Leptotrichia | *Leptotrichia hongkongensis* |
| Bacteria | Fusobacteriota | Fusobacteriia | Fusobacteriales | Leptotrichiaceae | Leptotrichia | *Leptotrichiamassiliensis* |
| Bacteria | Fusobacteriota | Fusobacteriia | Fusobacteriales | Leptotrichiaceae | Leptotrichia | *Leptotrichia shahii* |
| Bacteria | Fusobacteriota | Fusobacteriia | Fusobacteriales | Leptotrichiaceae | Leptotrichia | *Leptotrichia trevisanii* |
| Bacteria | Fusobacteriota | Fusobacteriia | Fusobacteriales | Leptotrichiaceae | Leptotrichia | *Leptotrichia wadei* |
| Bacteria | Proteobacteria | Gammaproteobacteria | Burkholderiales | Oxalobacteraceae | Massilia | *Massilia consociata* |
| Bacteria | Proteobacteria | Gammaproteobacteria | Burkholderiales | Oxalobacteraceae | Massilia | *Massilia haematophila* |
| Bacteria | Proteobacteria | Gammaproteobacteria | Burkholderiales | Oxalobacteraceae | Massilia | *Massilia oculi* |
| Bacteria | Proteobacteria | Gammaproteobacteria | Burkholderiales | Oxalobacteraceae | Massilia | *Massilia varians* |
| Bacteria | Firmicutes | Negativicutes | Veillonellales-Selenomonadales | Veillonellaceae | Megasphaera | *Megasphaera micronuciformis* |
| Bacteria | Proteobacteria | Alphaproteobacteria | Rhizobiales | Beijerinckiaceae | Microvirga | *Microvirga lotononidis* |
| Bacteria | Proteobacteria | Alphaproteobacteria | Rhizobiales | Beijerinckiaceae | Microvirga | *Microvirga subterranea* |
| Bacteria | Proteobacteria | Gammaproteobacteria | Pseudomonadales | Moraxellaceae | Moraxella | *Moraxella nonliquefaciens* |
| Bacteria | Bacteroidota | Bacteroidia | Sphingobacteriales | Sphingobacteriaceae | Mucilaginibacter | *Mucilaginibacter carri* |
| Bacteria | Firmicutes | Bacilli | Mycoplasmatales | Mycoplasmataceae | Mycoplasma | *Mycoplasma amphoriforme* |
| Bacteria | Firmicutes | Bacilli | Mycoplasmatales | Mycoplasmataceae | Mycoplasma | *Mycoplasma orale* |
| Bacteria | Firmicutes | Bacilli | Mycoplasmatales | Mycoplasmataceae | Mycoplasma | *Mycoplasma salivarium* |
| Bacteria | Proteobacteria | Gammaproteobacteria | Burkholderiales | Neisseriaceae | Neisseria | *Neisseria flavescens* |
| Bacteria | Proteobacteria | Gammaproteobacteria | Burkholderiales | Neisseriaceae | Neisseria | *Neisseria lactamica* |
| Bacteria | Proteobacteria | Gammaproteobacteria | Burkholderiales | Neisseriaceae | Neisseria | *Neisseria mucosa* |
| Bacteria | Proteobacteria | Gammaproteobacteria | Burkholderiales | Neisseriaceae | Neisseria | *Neisseria oralis* |
| Bacteria | Proteobacteria | Gammaproteobacteria | Burkholderiales | Neisseriaceae | Neisseria | *Neisseria pharyngis* |
| Bacteria | Proteobacteria | Gammaproteobacteria | Burkholderiales | Neisseriaceae | Neisseria | *Neisseria polysaccharea* |
| Bacteria | Proteobacteria | Gammaproteobacteria | Burkholderiales | Neisseriaceae | Neisseria | *Neisseria subflava* |
| Bacteria | Actinobacteriota | Actinobacteria | Micrococcales | Micrococcaceae | Nesterenkonia | *Nesterenkonia lacusekhoensis* |
| Bacteria | Actinobacteriota | Actinobacteria | Propionibacteriales | Nocardioidaceae | Nocardioides | *Nocardioides ganghwensis* |
| Bacteria | Actinobacteriota | Actinobacteria | Propionibacteriales | Nocardioidaceae | Nocardioides | *Nocardioides solisilvae* |
| Bacteria | Firmicutes | Bacilli | Bacillales | Bacillaceae | Oceanobacillus | *Oceanobacillus luteolus* |
| Bacteria | Firmicutes | Clostridia | Lachnospirales | Lachnospiraceae | Oribacterium | *Oribacterium asaccharolyticum* |
| Bacteria | Firmicutes | Clostridia | Lachnospirales | Lachnospiraceae | Oribacterium | *Oribacterium parvum* |
| Bacteria | Firmicutes | Clostridia | Lachnospirales | Lachnospiraceae | Oribacterium | *Oribacterium sinus* |
| Bacteria | Actinobacteriota | Actinobacteria | Micrococcales | Intrasporangiaceae | Ornithinimicrobium | *Ornithinimicrobium flavum* |
| Bacteria | Proteobacteria | Alphaproteobacteria | Rhodobacterales | Rhodobacteraceae | Paracoccus | *Paracoccus sanguinis* |
| Bacteria | Firmicutes | Bacilli | Lactobacillales | Lactobacillaceae | Pediococcus | *Pediococcus pentosaceus* |
| Bacteria | Firmicutes | Clostridia | Peptostreptococcales-Tissierellales | Peptostreptococcaceae | Peptoanaerobacter | *Peptoanaerobacter stomatis* |
| Bacteria | Bacteroidota | Bacteroidia | Bacteroidales | Bacteroidales Incertae Sedis | Phocaeicola | *Phocaeicola abscessus* |
| Bacteria | Firmicutes | Bacilli | Bacillales | Planococcaceae | Planomicrobium | *Planomicrobium okeanokoites* |
| Bacteria | Bacteroidota | Bacteroidia | Bacteroidales | Porphyromonadaceae | Porphyromonas | *Porphyromonas endodontalis* |
| Bacteria | Bacteroidota | Bacteroidia | Bacteroidales | Porphyromonadaceae | Porphyromonas | *Porphyromonas gingivalis* |
| Bacteria | Bacteroidota | Bacteroidia | Bacteroidales | Porphyromonadaceae | Porphyromonas | *Porphyromonas pasteri* |
| Bacteria | Bacteroidota | Bacteroidia | Bacteroidales | Porphyromonadaceae | Porphyromonas | *Porphyromonas uenonis* |
| Bacteria | Bacteroidota | Bacteroidia | Bacteroidales | Prevotellaceae | Prevotella | *Prevotella aurantiaca* |
| Bacteria | Bacteroidota | Bacteroidia | Bacteroidales | Prevotellaceae | Prevotella | *Prevotella baroniae* |
| Bacteria | Bacteroidota | Bacteroidia | Bacteroidales | Prevotellaceae | Prevotella | *Prevotella conceptionensis* |
| Bacteria | Bacteroidota | Bacteroidia | Bacteroidales | Prevotellaceae | Prevotella | *Prevotella dentalis* |
| Bacteria | Bacteroidota | Bacteroidia | Bacteroidales | Prevotellaceae | Prevotella | *Prevotella fusca* |
| Bacteria | Bacteroidota | Bacteroidia | Bacteroidales | Prevotellaceae | Prevotella | *Prevotella histicola* |
| Bacteria | Bacteroidota | Bacteroidia | Bacteroidales | Prevotellaceae | Prevotella | *Prevotella intermedia* |
| Bacteria | Bacteroidota | Bacteroidia | Bacteroidales | Prevotellaceae | Prevotella | *Prevotella jejuni* |
| Bacteria | Bacteroidota | Bacteroidia | Bacteroidales | Prevotellaceae | Prevotella | *Prevotella maculosa* |
| Bacteria | Bacteroidota | Bacteroidia | Bacteroidales | Prevotellaceae | Prevotella | *Prevotella melaninogenica* |
| Bacteria | Bacteroidota | Bacteroidia | Bacteroidales | Prevotellaceae | Prevotella | *Prevotella micans* |
| Bacteria | Bacteroidota | Bacteroidia | Bacteroidales | Prevotellaceae | Prevotella | *Prevotella nanceiensis* |
| Bacteria | Bacteroidota | Bacteroidia | Bacteroidales | Prevotellaceae | Prevotella | *Prevotella nigrescens* |
| Bacteria | Bacteroidota | Bacteroidia | Bacteroidales | Prevotellaceae | Prevotella | *Prevotella oris* |
| Bacteria | Bacteroidota | Bacteroidia | Bacteroidales | Prevotellaceae | Prevotella | *Prevotella pallens* |
| Bacteria | Bacteroidota | Bacteroidia | Bacteroidales | Prevotellaceae | Prevotella | *Prevotella saccharolytica* |
| Bacteria | Bacteroidota | Bacteroidia | Bacteroidales | Prevotellaceae | Prevotella | *Prevotella salivae* |
| Bacteria | Bacteroidota | Bacteroidia | Bacteroidales | Prevotellaceae | Prevotella | *Prevotella scopos* |
| Bacteria | Bacteroidota | Bacteroidia | Bacteroidales | Prevotellaceae | Prevotella | *Prevotella shahii* |
| Bacteria | Actinobacteriota | Actinobacteria | Micrococcales | Cellulomonadaceae | Pseudactinotalea | *Pseudactinotalea terrae* |
| Bacteria | Proteobacteria | Gammaproteobacteria | Pseudomonadales | Pseudomonadaceae | Pseudomonas | *Pseudomonas luteola* |
| Bacteria | Firmicutes | Clostridia | Eubacteriales | Eubacteriaceae | Pseudoramibacter | *Pseudoramibacter alactolyticus* |
| Bacteria | Proteobacteria | Gammaproteobacteria | Diplorickettsiales | Diplorickettsiaceae | Rickettsiella | *Rickettsiella isopodorum* |
| Bacteria | Proteobacteria | Alphaproteobacteria | Acetobacterales | Acetobacteraceae | Roseomonas | *Roseomonas ludipueritiae* |
| Bacteria | Actinobacteriota | Actinobacteria | Micrococcales | Micrococcaceae | Rothia | *Rothia dentocariosa* |
| Bacteria | Actinobacteriota | Actinobacteria | Micrococcales | Micrococcaceae | Rothia | *Rothia mucilaginosa* |
| Bacteria | Bacteroidota | Bacteroidia | Cytophagales | Hymenobacteraceae | Rufibacter | *Rufibacter immobilis* |
| Bacteria | Firmicutes | Bacilli | Staphylococcales | Staphylococcaceae | Salinicoccus | *Salinicoccus carnicancri* |
| Bacteria | Firmicutes | Negativicutes | Veillonellales-Selenomonadales | Selenomonadaceae | Selenomonas | *Selenomonas noxia* |
| Bacteria | Firmicutes | Negativicutes | Veillonellales-Selenomonadales | Selenomonadaceae | Selenomonas | *Selenomonas sputigena* |
| Bacteria | Proteobacteria | Alphaproteobacteria | Azospirillales | Azospirillaceae | Skermanella | *Skermanella aerolata* |
| Bacteria | Fusobacteriota | Fusobacteriia | Fusobacteriales | Leptotrichiaceae | Sneathia | *Sneathia sanguinegens* |
| Bacteria | Firmicutes | Bacilli | Erysipelotrichales | Erysipelotrichaceae | Solobacterium | *Solobacterium moorei* |
| Bacteria | Bacteroidota | Bacteroidia | Sphingobacteriales | Sphingobacteriaceae | Sphingobacterium | *Sphingobacterium multivorum* |
| Bacteria | Bacteroidota | Bacteroidia | Sphingobacteriales | Sphingobacteriaceae | Sphingobacterium | *Sphingobacterium siyangense* |
| Bacteria | Proteobacteria | Alphaproteobacteria | Sphingomonadales | Sphingomonadaceae | Sphingobium | *Sphingobium yanoikuyae* |
| Bacteria | Proteobacteria | Alphaproteobacteria | Sphingomonadales | Sphingomonadaceae | Sphingomonas | *Sphingomonas desiccabilis* |
| Bacteria | Proteobacteria | Alphaproteobacteria | Sphingomonadales | Sphingomonadaceae | Sphingomonas | *Sphingomonas humi* |
| Bacteria | Proteobacteria | Alphaproteobacteria | Sphingomonadales | Sphingomonadaceae | Sphingomonas | *Sphingomonas melonis* |
| Bacteria | Proteobacteria | Alphaproteobacteria | Sphingomonadales | Sphingomonadaceae | Sphingomonas | *Sphingomonas yunnanensis* |
| Bacteria | Firmicutes | Bacilli | Staphylococcales | Staphylococcaceae | Staphylococcus | *Staphylococcus aureus* |
| Bacteria | Firmicutes | Bacilli | Staphylococcales | Staphylococcaceae | Staphylococcus | *Staphylococcus epidermidis* |
| Bacteria | Firmicutes | Bacilli | Staphylococcales | Staphylococcaceae | Staphylococcus | *Staphylococcus hominis* |
| Bacteria | Firmicutes | Bacilli | Staphylococcales | Staphylococcaceae | Staphylococcus | *Staphylococcus pasteuri* |
| Bacteria | Proteobacteria | Gammaproteobacteria | Xanthomonadales | Xanthomonadaceae | Stenotrophomonas | *Stenotrophomonas maltophilia* |
| Bacteria | Firmicutes | Clostridia | Lachnospirales | Lachnospiraceae | Stomatobaculum | *Stomatobaculum longum* |
| Bacteria | Fusobacteriota | Fusobacteriia | Fusobacteriales | Leptotrichiaceae | Streptobacillus | *Streptobacillus hongkongensis* |
| Bacteria | Firmicutes | Bacilli | Lactobacillales | Streptococcaceae | Streptococcus | *Streptococcus anginosus* |
| Bacteria | Firmicutes | Bacilli | Lactobacillales | Streptococcaceae | Streptococcus | *Streptococcus cristatus* |
| Bacteria | Firmicutes | Bacilli | Lactobacillales | Streptococcaceae | Streptococcus | *Streptococcus gordonii* |
| Bacteria | Firmicutes | Bacilli | Lactobacillales | Streptococcaceae | Streptococcus | *Streptococcus lactarius* |
| Bacteria | Firmicutes | Bacilli | Lactobacillales | Streptococcaceae | Streptococcus | *Streptococcus mitis* |
| Bacteria | Firmicutes | Bacilli | Lactobacillales | Streptococcaceae | Streptococcus | *Streptococcus mutans* |
| Bacteria | Firmicutes | Bacilli | Lactobacillales | Streptococcaceae | Streptococcus | *Streptococcus oralis* |
| Bacteria | Firmicutes | Bacilli | Lactobacillales | Streptococcaceae | Streptococcus | *Streptococcus parasanguinis* |
| Bacteria | Firmicutes | Bacilli | Lactobacillales | Streptococcaceae | Streptococcus | *Streptococcus pseudopneumoniae* |
| Bacteria | Firmicutes | Bacilli | Lactobacillales | Streptococcaceae | Streptococcus | *Streptococcus salivarius* |
| Bacteria | Firmicutes | Bacilli | Lactobacillales | Streptococcaceae | Streptococcus | *Streptococcus sinensis* |
| Bacteria | Bacteroidota | Bacteroidia | Bacteroidales | Tannerellaceae | Tannerella | *Tannerella forsythia* |
| Bacteria | Spirochaetota | Spirochaetia | Spirochaetales | Spirochaetaceae | Treponema | *Treponema amylovorum* |
| Bacteria | Spirochaetota | Spirochaetia | Spirochaetales | Spirochaetaceae | Treponema | *Treponema denticola* |
| Bacteria | Spirochaetota | Spirochaetia | Spirochaetales | Spirochaetaceae | Treponema | *Treponema lecithinolyticum* |
| Bacteria | Spirochaetota | Spirochaetia | Spirochaetales | Spirochaetaceae | Treponema | *Treponema maltophilum* |
| Bacteria | Spirochaetota | Spirochaetia | Spirochaetales | Spirochaetaceae | Treponema | *Treponema medium* |
| Bacteria | Spirochaetota | Spirochaetia | Spirochaetales | Spirochaetaceae | Treponema | *Treponema parvum* |
| Bacteria | Spirochaetota | Spirochaetia | Spirochaetales | Spirochaetaceae | Treponema | *Treponema pectinovorum* |
| Bacteria | Spirochaetota | Spirochaetia | Spirochaetales | Spirochaetaceae | Treponema | *Treponema putidum* |
| Bacteria | Spirochaetota | Spirochaetia | Spirochaetales | Spirochaetaceae | Treponema | *Treponema socranskii* |
| Bacteria | Spirochaetota | Spirochaetia | Spirochaetales | Spirochaetaceae | Treponema | *Treponema vincentii* |
| Bacteria | Firmicutes | Bacilli | Alicyclobacillales | Alicyclobacillaceae | Tumebacillus | *Tumebacillus luteolus* |
| Bacteria | Firmicutes | Negativicutes | Veillonellales-Selenomonadales | Veillonellaceae | Veillonella | *Veillonella atypica* |
| Bacteria | Firmicutes | Negativicutes | Veillonellales-Selenomonadales | Veillonellaceae | Veillonella | *Veillonella dispar* |
| Bacteria | Firmicutes | Negativicutes | Veillonellales-Selenomonadales | Veillonellaceae | Veillonella | *Veillonella massiliensis* |
| Bacteria | Firmicutes | Negativicutes | Veillonellales-Selenomonadales | Veillonellaceae | Veillonella | *Veillonella parvula* |
| Bacteria | Firmicutes | Negativicutes | Veillonellales-Selenomonadales | Veillonellaceae | Veillonella | *Veillonella rogosae* |
| Bacteria | Firmicutes | Negativicutes | Veillonellales-Selenomonadales | Veillonellaceae | Veillonella | *Veillonella tobetsuensis* |
| Bacteria | Firmicutes | Bacilli | Bacillales | Bacillaceae | Virgibacillus | *Virgibacillus siamensis* |
| Bacteria | Firmicutes | Bacilli | Lactobacillales | Leuconostocaceae | Weissella | *Weissella cibaria* |

**Supplementary Table 2:** Overview of the 20 Amplicon Sequence Variant (ASV) used in this study

| **Bacteria** | **Sequence** |
| --- | --- |
| Neisseria meningit*idis* ASV -269, 16S rRNA gene | GGAATTTTGGACAATGGGCGCAAGCCTGATCCAGCCATGCCGCGTGTCTGAAGAAGGCCTTCGGGTTGTAAAGGACTTTTGTCAGGGAAGAAAAGGCTGTTGCTAATACCAGCGGCCGATGACGGTACCTGAAGAATAAGCACCGGCTAACTACGTGCCAGCAGCCGCGGTAATACGTAGGGTGCGAGCGTTAATCGGAATTACTGGGCGTAAAGCGGGCGCAGACGGTTACTTAAGCAGGATGTGAAATCCCCGGGCTCAACCCGGGAACTGCGTTCTGAACTGGGTGACTCGAGTGTGTCAGAGGGAGGTAGAATTCCACGTGTAGCAGTGAAATGCGTAGAGATGTGGAGGAATACCGATGGCGAAGGCAGCCTCCTGGGACAACACTGACGTTCATGCCCGAAAGCGTGGGTAGCAAACAG |
| *Streptococcus pneumoniae* ASV-1034, 16S rRNA gene | GGAATCTTCGGCAATGGACGGAAGTCTGACCGAGCAACGCCGCGTGAGTGAAGAAGGTTTTCGGATCGTAAAGCTCTGTTGTAAGAGAAGAACGAGTGTGAGAGTGGAAAGTTCACGCTGTGACGGTATCTTACCAGAAAGGGACGGCTAACTACGTGCCAGCAGCCGCGGTAATACGTAGGTCCCGAGCGTTGTCCGGATTTATTGGGCGTAAAGCGAGCGCAGGCGGTTAGATAAGTCTGAAGTTAAAGGCTGTGGCTTAACCATAGTAGGCTTTGGAAACTGTTTAACTTGAGTGCAAGAGGGGAGAGTGGAATTCCATGTGTAGCGGTGAAATGCGTAGATATATGGAGGAACACCGGTGGCGAAAGCGGCTCTCTGGCTTGTAACTGACGCTGAGGCTCGAAAGCGTGGGGAGCAAACAG |
| *Streptococcus pneumoniae* ASV-2112, 16S rRNA gene | GGAATCTTCGGCAATGGACGAAAGTCTGACCGAGCAACGCCGCGTGAGTGAAGAAGGTTTTCGGATCGTAAAGCTCTGTTGTAAGAGAAGAACGAGTGTGAGAGTGGAAAGTTCACACTGTGACGGTATCTTACCAGAAAGGGACGGCTAACTACGTGCCAGCAGCCGCGGTAATACGTAGGTCCCGAGCGTTGTCCGGATTTATTGGGCGTAAAGCGAGCGCAGGCGGTTAGATAAGTCTGAAGTTAAAGGCTGTGGCTTAACCATAGTAGGCTTTGGAAACTGTTTAACTTGAGTGCAAGAGGGGAGAGTGGAATTCCATGTGTAGCGGTGAAATGCGTAGATATATGGAGGAACACCGGTGGCGAAAGCGGCTCTCTGGCTTGTAACTGACGCTGAGGCTCGAAAGCGTGGGGAGCAAACAG |
| *Haemophilus influenzae* ASV-19, 16S rRNA gene | GGAATATTGCGCAATGGGGGCAACCCTGACGCAGCCATGCCGCGTGAATGAAGAAGGCCTTCGGGTTGTAAAGTTCTTTCGGTATTGAGGAAGGTTGATGTGTTAATAGTACATCAAATTGACGTTAAATACAGAAGAAGCACCGGCTAACTCCGTGCCAGCAGCCGCGGTAATACGGAGGGTGCGAGCGTTAATCGGAATAACTGGGCGTAAAGGGCACGCAGGCGGTTATTTAAGTGAGGTGTGAAAGCCCCGGGCTTAACCTGGGAATTGCATTTCAGACTGGGTAACTAGAGTACTTTAGGGAGGGGTAGAATTCCACGTGTAGCGGTGAAATGCGTAGAGATGTGGAGGAATACCGAAGGCGAAGGCAGCCCCTTGGGAATGTACTGACGCTCATGTGCGAAAGCGTGGGGAGCAAACAG |
| *Haemophilus influenzae* ASV-23, 16S rRNA gene | GGAATATTGCGCAATGGGGGCAACCCTGACGCAGCCATGCCGCGTGAATGAAGAAGGCCTTCGGGTTGTAAAGTTCTTTCGGTATTGAGGAAGGTTGATGTGTTAATAGTACATCAAATTGACGTTAAATACAGAAGAAGCACCGGCTAACTCCGTGCCAGCAGCCGCGGTAATACGGAGGGTGCGAGCGTTAATCGGAATAACTGGGCGTAAAGGGCACGCAGGCGGTTATTTAAGTGAGGTGTGAAAGCCCCGGGCTTAACCTGGGAATTGCATTTCAGACTGGGTAACTAGAGTACTTTAGGGAGGGGTAGAATTCCACGTGTAGCGGTGAAATGCGTAGAGATGTGGAGGAATACCGAAGGCGAAGGCAGCCCCTTGGGAATGTACTGACGCTCATGTGCGAAAGCGTGGGGAGCAAACAG |
| *Haemophilus influenzae* ASV-46, 16S rRNA gene | GGAATATTGCGCAATGGGGGCAACCCTGACGCAGCCATGCCGCGTGAATGAAGAAGGCCTTCGGGTTGTAAAGTTCTTTCGGTATTGAGGAAGGTTGATGTGTTAATAGTACATCAAATTGACGTTAAATACAGAAGAAGCACCGGCTAACTCCGTGCCAGCAGCCGCGGTAATACGGAGGGTGCGAGCGTTAATCGGAATAACTGGGCGTAAAGGGCACGCAGGCGGTTATTTAAGTGAGGTGTGAAAGCCCCGGGCTTAACCTGGGAATTGCATTTCAGACTGGGTAACTAGAGTACTTTAGGGAGGGGTAGAATTCCACGTGTAGCGGTGAAATGCGTAGAGATGTGGAGGAATACCGAAGGCGAAGGCAGCCCCTTGGGAATGTACTGACGCTCATGTGCGAAAGCGTGGGGAGCAAACAG |
| *Haemophilus influenzae* ASV-52, 16S rRNA gene | GGAATATTGCGCAATGGGGGCAACCCTGACGCAGCCATGCCGCGTGAATGAAGAAGGCCTTCGGGTTGTAAAGTTCTTTCGGTATTGAGGAAGGTTGATGTGTTAATAGTACATCAAATTGACGTTAAATACAGAAGAAGCACCGGCTAACTCCGTGCCAGCAGCCGCGGTAATACGGAGGGTGCGAGCGTTAATCGGAATAACTGGGCGTAAAGGGCACGCAGGCGGTTATTTAAGTGAGGTGTGAAAGCCCCGGGCTTAACCTGGGAATTGCATTTCAGACTGGGTAACTAGAGTACTTTAGGGAGGGGTAGAATTCCACGTGTAGCGGTGAAATGCGTAGAGATGTGGAGGAATACCGAAGGCGAAGGCAGCCCCTTGGGAATGTACTGACGCTCATGTGCGAAAGCGTGGGGAGCAAACAG |
| *Haemophilus influenzae* ASV-84, 16S rRNA gene | GGAATATTGCGCAATGGGGGCAACCCTGACGCAGCCATGCCGCGTGAATGAAGAAGGCCTTCGGGTTGTAAAGTTCTTTCGGTATTGAGGAAGGTTGATGTGTTAATAGTACATCAAATTGACGTTAAATACAGAAGAAGCACCGGCTAACTCCGTGCCAGCAGCCGCGGTAATACGGAGGGTGCGAGCGTTAATCGGAATAACTGGGCGTAAAGGGCACGCAGGCGGTTATTTAAGTGAGGTGTGAAAGCCCCGGGCTTAACCTGGGAATTGCATTTCAGACTGGGTAACTAGAGTACTTTAGGGAGGGGTAGAATTCCACGTGTAGCGGTGAAATGCGTAGAGATGTGGAGGAATACCGAAGGCGAAGGCAGCCCCTTGGGAATGTACTGACGCTCATGTGCGAAAGCGTGGGGAGCAAACAG |
| *Haemophilus influenzae* ASV-86, 16S rRNA gene | GGAATATTGCGCAATGGGGGCAACCCTGACGCAGCCATGCCGCGTGAATGAAGAAGGCCTTCGGGTTGTAAAGTTCTTTCGGTATTGAGGAAGGTTGATGTGTTAATAGTACATCAAATTGACGTTAAATACAGAAGAAGCACCGGCTAACTCCGTGCCAGCAGCCGCGGTAATACGGAGGGTGCGAGCGTTAATCGGAATAACTGGGCGTAAAGGGCACGCAGGCGGTTATTTAAGTGAGGTGTGAAAGCCCCGGGCTTAACCTGGGAATTGCATTTCAGACTGGGTAACTAGAGTACTTTAGGGAGGGGTAGAATTCCACGTGTAGCGGTGAAATGCGTAGAGATGTGGAGGAATACCGAAGGCGAAGGCAGCCCCTTGGGAATGTACTGACGCTCATGTGCGAAAGCGTGGGGAGCAAACAG |
| *Haemophilus influenzae* ASV-112, 16S rRNA gene | GGAATATTGCGCAATGGGGGCAACCCTGACGCAGCCATGCCGCGTGAATGAAGAAGGCCTTCGGGTTGTAAAGTTCTTTCGGTATTGAGGAAGGTTGATGTGTTAATAGTACATCAAATTGACGTTAAATACAGAAGAAGCACCGGCTAACTCCGTGCCAGCAGCCGCGGTAATACGGAGGGTGCGAGCGTTAATCGGAATAACTGGGCGTAAAGGGCACGCAGGCGGTTATTTAAGTGAGGTGTGAAAGCCCCGGGCTTAACCTGGGAATTGCATTTCAGACTGGGTAACTAGAGTACTTTAGGGAGGGGTAGAATTCCACGTGTAGCGGTGAAATGCGTAGAGATGTGGAGGAATACCGAAGGCGAAGGCAGCCCCTTGGGAATGTACTGACGCTCATGTGCGAAAGCGTGGGGAGCAAACAG |
| *Haemophilus influenzae* ASV-172, 16S rRNA gene | GGAATATTGCGCAATGGGGGCAACCCTGACGCAGCCATGCCGCGTGAATGAAGAAGGCCTTCGGGTTGTAAAGTTCTTTCGGTATTGAGGAAGGTTGATGTGTTAATAGTACATCAAATTGACGTTAAATACAGAAGAAGCACCGGCTAACTCCGTGCCAGCAGCCGCGGTAATACGGAGGGTGCGAGCGTTAATCGGAATAACTGGGCGTAAAGGGCACGCAGGCGGTTATTTAAGTGAGGTGTGAAAGCCCCGGGCTTAACCTGGGAATTGCATTTCAGACTGGGTAACTAGAGTACTTTAGGGAGGGGTAGAATTCCACGTGTAGCGGTGAAATGCGTAGAGATGTGGAGGAATACCGAAGGCGAAGGCAGCCCCTTGGGAATGTACTGACGCTCATGTGCGAAAGCGTGGGGAGCAAACAG |
| *Haemophilus influenzae* ASV-192, 16S rRNA gene | GGAATATTGCGCAATGGGGGCAACCCTGACGCAGCCATGCCGCGTGAATGAAGAAGGCCTTCGGGTTGTAAAGTTCTTTCGGTATTGAGGAAGGTTGATGTGTTAATAGTACATCAAATTGACGTTAAATACAGAAGAAGCACCGGCTAACTCCGTGCCAGCAGCCGCGGTAATACGGAGGGTGCGAGCGTTAATCGGAATAACTGGGCGTAAAGGGCACGCAGGCGGTTATTTAAGTGAGGTGTGAAAGCCCCGGGCTTAACCTGGGAATTGCATTTCAGACTGGGTAACTAGAGTACTTTAGGGAGGGGTAGAATTCCACGTGTAGCGGTGAAATGCGTAGAGATGTGGAGGAATACCGAAGGCGAAGGCAGCCCCTTGGGAATGTACTGACGCTCATGTGCGAAAGCGTGGGGAGCAAACAG |
| *Haemophilus influenzae* ASV-247, 16S rRNA gene | GGAATATTGCGCAATGGGGGCAACCCTGACGCAGCCATGCCGCGTGAATGAAGAAGGCCTTCGGGTTGTAAAGTTCTTTCGGTATTGAGGAAGGTTGATGTGTTAATAGTACATCAAATTGACGTTAAATACAGAAGAAGCACCGGCTAACTCCGTGCCAGCAGCCGCGGTAATACGGAGGGTGCGAGCGTTAATCGGAATAACTGGGCGTAAAGGGCACGCAGGCGGTTATTTAAGTGAGGTGTGAAAGCCCCGGGCTTAACCTGGGAATTGCATTTCAGACTGGGTAACTAGAGTACTTTAGGGAGGGGTAGAATTCCACGTGTAGCGGTGAAATGCGTAGAGATGTGGAGGAATACCGAAGGCGAAGGCAGCCCCTTGGGAATGTACTGACGCTCATGTGCGAAAGCGTGGGGAGCAAACAG |
| *Haemophilus influenzae* ASV-312, 16S rRNA gene | GGAATATTGCGCAATGGGGGCAACCCTGACGCAGCCATGCCGCGTGAATGAAGAAGGCCTTCGGGTTGTAAAGTTCTTTCGGTATTGAGGAAGGTTGATGTGTTAATAGTACATCAAATTGACGTTAAATACAGAAGAAGCACCGGCTAACTCCGTGCCAGCAGCCGCGGTAATACGGAGGGTGCGAGCGTTAATCGGAATAACTGGGCGTAAAGGGCACGCAGGCGGTTATTTAAGTGAGGTGTGAAAGCCCCGGGCTTAACCTGGGAATTGCATTTCAGACTGGGTAACTAGAGTACTTTAGGGAGGGGTAGAATTCCACGTGTAGCGGTGAAATGCGTAGAGATGTGGAGGAATACCGAAGGCGAAGGCAGCCCCTTGGGAATGTACTGACGCTCATGTGCGAAAGCGTGGGGAGCAAACAG |
| *Haemophilus influenzae* ASV-360, 16S rRNA gene | GGAATATTGCGCAATGGGGGCAACCCTGACGCAGCCATGCCGCGTGAATGAAGAAGGCCTTCGGGTTGTAAAGTTCTTTCGGTATTGAGGAAGGTTGATGTGTTAATAGTACATCAAATTGACGTTAAATACAGAAGAAGCACCGGCTAACTCCGTGCCAGCAGCCGCGGTAATACGGAGGGTGCGAGCGTTAATCGGAATAACTGGGCGTAAAGGGCACGCAGGCGGTTATTTAAGTGAGGTGTGAAAGCCCCGGGCTTAACCTGGGAATTGCATTTCAGACTGGGTAACTAGAGTACTTTAGGGAGGGGTAGAATTCCACGTGTAGCGGTGAAATGCGTAGAGATGTGGAGGAATACCGAAGGCGAAGGCAGCCCCTTGGGAATGTACTGACGCTCATGTGCGAAAGCGTGGGGAGCAAACAG |
| *Haemophilus influenzae* ASV-389, 16S rRNA gene | GGAATATTGCGCAATGGGGGCAACCCTGACGCAGCCATGCCGCGTGAATGAAGAAGGCCTTCGGGTTGTAAAGTTCTTTCGGTATTGAGGAAGGTTGATGTGTTAATAGTACATCAAATTGACGTTAAATACAGAAGAAGCACCGGCTAACTCCGTGCCAGCAGCCGCGGTAATACGGAGGGTGCGAGCGTTAATCGGAATAACTGGGCGTAAAGGGCACGCAGGCGGTTATTTAAGTGAGGTGTGAAAGCCCCGGGCTTAACCTGGGAATTGCATTTCAGACTGGGTAACTAGAGTACTTTAGGGAGGGGTAGAATTCCACGTGTAGCGGTGAAATGCGTAGAGATGTGGAGGAATACCGAAGGCGAAGGCAGCCCCTTGGGAATGTACTGACGCTCATGTGCGAAAGCGTGGGGAGCAAACAG |
| *Haemophilus influenzae* ASV-432, 16S rRNA gene | GGAATATTGCGCAATGGGGGCAACCCTGACGCAGCCATGCCGCGTGAATGAAGAAGGCCTTCGGGTTGTAAAGTTCTTTCGGTATTGAGGAAGGTTGATGTGTTAATAGTACATCAAATTGACGTTAAATACAGAAGAAGCACCGGCTAACTCCGTGCCAGCAGCCGCGGTAATACGGAGGGTGCGAGCGTTAATCGGAATAACTGGGCGTAAAGGGCACGCAGGCGGTTATTTAAGTGAGGTGTGAAAGCCCCGGGCTTAACCTGGGAATTGCATTTCAGACTGGGTAACTAGAGTACTTTAGGGAGGGGTAGAATTCCACGTGTAGCGGTGAAATGCGTAGAGATGTGGAGGAATACCGAAGGCGAAGGCAGCCCCTTGGGAATGTACTGACGCTCATGTGCGAAAGCGTGGGGAGCAAACAG |
| *Haemophilus influenzae* ASV-466, 16S rRNA gene | GGAATATTGCGCAATGGGGGCAACCCTGACGCAGCCATGCCGCGTGAATGAAGAAGGCCTTCGGGTTGTAAAGTTCTTTCGGTATTGAGGAAGGTTGATGTGTTAATAGTACATCAAATTGACGTTAAATACAGAAGAAGCACCGGCTAACTCCGTGCCAGCAGCCGCGGTAATACGGAGGGTGCGAGCGTTAATCGGAATAACTGGGCGTAAAGGGCACGCAGGCGGTTATTTAAGTGAGGTGTGAAAGCCCCGGGCTTAACCTGGGAATTGCATTTCAGACTGGGTAACTAGAGTACTTTAGGGAGGGGTAGAATTCCACGTGTAGCGGTGAAATGCGTAGAGATGTGGAGGAATACCGAAGGCGAAGGCAGCCCCTTGGGAATGTACTGACGCTCATGTGCGAAAGCGTGGGGAGCAAACAG |
| *Haemophilus influenzae* ASV-508, 16S rRNA gene | GGAATATTGCGCAATGGGGGCAACCCTGACGCAGCCATGCCGCGTGAATGAAGAAGGCCTTCGGGTTGTAAAGTTCTTTCGGTATTGAGGAAGGTTGATGTGTTAATAGTACATCAAATTGACGTTAAATACAGAAGAAGCACCGGCTAACTCCGTGCCAGCAGCCGCGGTAATACGGAGGGTGCGAGCGTTAATCGGAATAACTGGGCGTAAAGGGCACGCAGGCGGTTATTTAAGTGAGGTGTGAAAGCCCCGGGCTTAACCTGGGAATTGCATTTCAGACTGGGTAACTAGAGTACTTTAGGGAGGGGTAGAATTCCACGTGTAGCGGTGAAATGCGTAGAGATGTGGAGGAATACCGAAGGCGAAGGCAGCCCCTTGGGAATGTACTGACGCTCATGTGCGAAAGCGTGGGGAGCAAACAG |
| *Haemophilus influenzae* ASV-1466, 16S rRNA gene | GGAATATTGCGCAATGGGGGCAACCCTGACGCAGCCATGCCGCGTGAATGAAGAAGGCCTTCGGGTTGTAAAGTTCTTTCGGTATTGAGGAAGGTTGATGTGTTAATAGTACATCAAATTGACGTTAAATACAGAAGAAGCACCGGCTAACTCCGTGCCAGCAGCCGCGGTAATACGGAGGGTGCGAGCGTTAATCGGAATAACTGGGCGTAAAGGGCACGCAGGCGGTTATTTAAGTGAGGTGTGAAAGCCCCGGGCTTAACCTGGGAATTGCATTTCAGACTGGGTAACTAGAGTACTTTAGGGAGGGGTAGAATTCCACGTGTAGCGGTGAAATGCGTAGAGATGTGGAGGAATACCGAAGGCGAAGGCAGCCCCTTGGGAATGTACTGACGCTCATGTGCGAAAGCGTGGGGAGCAAACAG |

**Supplementary Figure 1** : Distribution of per-sample read depth using filtered reads. **A** Sequencing depth per sample. **B** Signal to background ratio. **C** Read depth density distribution. Filtered reads were used to remove low-quality sequences. Samples had a median read depth of 124,656, exceeding that of controls (median 90,402).

**Supplementary Figure 2**: Maximum-likelihood molecular phylogenetic analysis of Neisseria meningitidis Amplicon Sequence Variant based on 16S rRNA gene sequences. Sequences generated in this study are shown in bold.
